# Loci and Natural Alleles for Low-Nitrogen-Induced Growth Response Revealed by the Genome-Wide Association Study Analysis in Rice (*Oryza sativa* L.)

**DOI:** 10.3389/fpls.2021.770736

**Published:** 2021-11-05

**Authors:** Yang Lv, Jie Ma, Yueying Wang, Quan Wang, Xueli Lu, Haitao Hu, Qian Qian, Longbiao Guo, Lianguang Shang

**Affiliations:** ^1^Rice Research Institute, Shenyang Agricultural University, Shenyang, China; ^2^State Key Laboratory of Rice Biology, China National Rice Research Institute, Hangzhou, China; ^3^Shenzhen Branch, Guangdong Laboratory of Lingnan Modern Agriculture, Genome Analysis Laboratory of the Ministry of Agriculture and Rural Affairs, Agricultural Genomics Institute at Shenzhen, Chinese Academy of Agricultural Sciences, Shenzhen, China

**Keywords:** rice, GWAS, low-nitrogen-induced growth response, haplotype analysis, gene mining

## Abstract

Nitrogen is essential for plant growth and yield, and it is, therefore, crucial to increase the nitrogen-use efficiency (NUE) of crop plants in fields. In this study, we measured four major low-nitrogen-induced growth response (LNGR) agronomic traits (i.e., plant height, tiller number, chlorophyll content, and leaf length) of the 225-rice-variety natural population from the Rice 3K Sequencing Project across normal nitrogen (NN) and low nitrogen (LN) environments. The LNGR phenotypic difference between NN and LN levels was used for gene analysis using a genome-wide association study (GWAS) combined with 111,205 single-nucleotide polymorphisms (SNPs) from the available sequenced data from the 3K project. We obtained a total of 56 significantly associated SNPs and 4 candidate genes for 4 LNGR traits. Some loci were located in the candidate regions, such as *MYB61*, *OsOAT*, and *MOC2*. To further study the role of candidate genes, we conducted haplotype analyses to identify the elite germplasms. Moreover, several other plausible candidate genes encoding LN-related or NUE proteins were worthy of mining. Our study provides novel insight into the genetic control of LNGR and further reveals some related novel haplotypes and potential genes with phenotypic variation in rice.

## Introduction

Nitrogen (N) is an essential macronutrient element for plant growth. Plants take up N from the soil mainly in the form of nitrate (NO_3_^–^) and/or ammonium (NH_4_^+^) (Wang et al., 2012; Fan et al., 2016). In crops, the demand for nitrogen is in accordance with its importance as the basic constituent of nucleotides, amino acids, and chlorophyll (Zhang M. et al., 2021). Nitrogen utilization involves multiple limiting factors, mainly including the varieties and the amount of nitrogen fertilizer, the method of nitrogen fertilizer application, and the expression of nitrogen genes (Ju et al., 2009; Yang et al., 2015). However, some elite rice germplasm can be harvested and produce the same biomass and grain yield under low N (LN) fertilizers, as compared with high N levels. This phenomenon is called low nitrogen-use efficiency (NUE), and thus, it mitigates detrimental effects on ecosystems (Good et al., 2004; Zhang X. et al., 2015). It is critically important to understand the molecular mechanisms of low-N-induced growth response (LNGR) and to develop crop varieties with improved NUE (Fan et al., 2017; Du et al., 2021; Yu et al., 2021).

The NUE is a complex trait controlled by multiple essential genes and quantitative trait loci (QTLs). Nitrogen-use efficiency is closely related to agronomic traits, such as plant height, tiller number, chlorophyll content, and yield, which can be markedly affected by N application in rice. In recent years, several NUE-related genes and loci have been identified by mutants and QTL mapping, targeting various components of nitrogen metabolism and potential regulators of NUE. *DENSE AND ERECT PANICLES 1* (*DEP1*) is a major typical QTL that controls rice yield traits, and its allele, *DEP1-1*, affects rice NUE. The dominant allele is a nitrogen-insensitive vegetative growth mutation that increases nitrogen uptake and assimilation, resulting in improved harvest index and grain yield at moderate levels of nitrogen fertilization (Sun et al., 2014, 2018).

Allelic variants for the nitrate-transporter gene *NRT1.1B* display distinctive divergence between two subspecies of rice, namely, *indica* and *japonica*, which show the difference in NO_3_^–^ utilization (Hu et al., 2015). Notably, *ARE1* (for *abc1 PEPRESSOR1*) is prominent due to its enhanced NUE under nitrogen-limiting conditions (Wang et al., 2018a). Subsequently, *grain number*, *plant height*, *and heading date7* (*Ghd7*) confer tolerance to LN, in order to positively regulate nitrogen utilization (Good et al., 2004; Saito et al., 2019; Wang et al., 2021). Additionally, the allelic variation in *OsNR2* (Gao Z. et al., 2019) caused differences in the NO_3_^–^ assimilation capacity and NUE of the *indica* and *japonica* subspecies. Moreover, it has been reported that other transcription factors, such as *OsGRF4* (Li et al., 2018), *OsTCP19* (Liu Y. et al., 2021), *OsNLP4* (Yu et al., 2021), *OsCCA1* (Zhang S. et al., 2021), *MYB61* (Gao Y. et al., 2020), and *OsNAC42* (Tang et al., 2019), participate in nitrogen utilization regulation. The molecular mechanisms of NUE between diverse rice varieties are still worthy of intensive exploration.

Increasing evidence has revealed that N supply has a significant impact on plant growth and development (McAllister et al., 2012; Chen et al., 2016). However, the conventional approach of linkage mapping has been used to identify potential genes with phenotypic variation, and even when this approach was limited to a biparental experimental population, it was time-consuming (Liu Z. et al., 2016; Wei et al., 2021).

Genome-wide association study (GWAS) is a high-resolution method for analyzing complex genetic traits in plants (Liu N. et al., 2016; Gao H. et al., 2020). Association mapping simultaneously reveals multiple loci of candidate genes with important agronomic traits. In this study, based on the genomic database and bioinformatics analysis, we carried out GWAS with 225 natural varieties of rice from the Rice 3K Sequencing Project with 4 LNGR agronomic phenotypes and analyzed their differences between normal nitrogen (NN) and LN conditions. We subsequently identified 56 LN-induced loci and 4 candidate genes for further analysis.

## Materials and Methods

### Plant Materials

A set of 225 natural varieties of rice were collected and selected from the Rice 3K Project (Supplementary Table 1), most of which belonged to the four genetic groups. The subpopulations present in this study were estimated using PowerMarker software. All varieties were stored at the China National Rice Research Institute (CNRRI, Hangzhou City, Zhejiang Province, China). All the materials were grown in the experimental field of the Agricultural Genomics Institute at Shenzhen (AGIS, Guangdong Province, China) over the cropping seasons (2020) and with a randomized complete block design.

### Statistical Analysis of Phenotypic Variation

We measured the LN-related agronomic traits of the soil and plant analyzer development value (SV), leaf length (LL), plant height (PH), and tiller number (TN) of the 225 rice varieties planted where there were NN levels (180 kg^⋅^hm^–2^) and LN levels (90 kg^⋅^hm^–2^). There were 20 and 18 cm between rows and individuals, respectively. Additionally, 10 plants were randomly selected from each variety at maturity. For field measurement, the flag leaf became the target of the SV and LL. We calculated the four-trait difference between NN and LN conditions as follows: (the NN phenotype value) – (the LN phenotype value of the same material).

The descriptive statistical analysis was performed in R 4.0.3 software. Based on the phenotypic data, four traits were validated for the analysis of correlation by GraphPad Prism.9 software.

### Association Analysis

The genotype data information released from the Rice 3K Sequencing Project (Wang et al., 2018c)^1^ was downloaded, and the association analysis was determined using EMMAX software (Segura et al., 2013). A total of 111,205 single-nucleotide polymorphisms (SNPs) were filtered with a missing rate < 0.4 and a minimum allele frequency (MAF) > 0.05 (Kang et al., 2010). A population structure correction was made by Admixture_linux-1.3.0 (Alexander and Lange, 2011) for the GWAS analysis.

### Candidate Gene

To present clearer association analyses and subsequent results, the significance level of SNPs was set at *P* ≤ 10^–5^. Simultaneously, in accordance with linkage disequilibrium (LD) decay values, we examined the candidate genes from ± 100 kb (i.e., 100 kb upstream and 100 kb downstream) of the significant site (Zhang C. et al., 2018). In this study, gene functional annotations and haplotype analysis were performed by the Rice Genome Annotation Project Database.^2^

### Phylogenetic Analyses

We used the PowerMarker (Liu and Muse, 2005) software to first estimated genetic diversity and distance measures of 225 natural rice varieties. We identified the varieties of structure and infer the classification of subpopulations among the individual varieties by using the model-based program POPGENE (Liu N. et al., 2016). We finally used R 4.0.3 software to statistically analyze the genetic relationship between subpopulations.

## Results

### Phenotyping of Four Low-N-Induced Agronomic Traits

**FIGURE 1:**
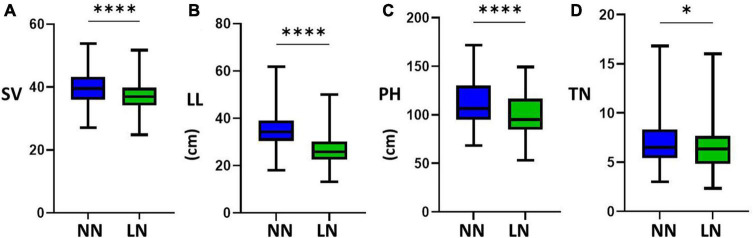
Boxplots of the mean amounts of four nitrogen-use efficiency (NUE)-related agronomic traits of different accessions under the normal nitrogen (NN) level and the low nitrogen (LN) level. Phenotypic traits of **(A)** soil and plant analyzer development value (SV), **(B)** leaf length (LL), **(C)** plant height (PH), and **(D)** tiller number (TN) (****P < 0.0001; *P < 0.05).

The four LN-related agronomic traits of SV, LL, PH, and TN of the 225 rice varieties with the representative LN-related phenotypes exhibited obvious differences in NN level and LN level (Figure 1). By evaluating the statistics data, we found a broad phenotypic variation of the NUE-related agronomic traits of the natural rice population, indicating that the LN-induced phenotypic data were reliable for further genetic analyses. The correlations of the mean amount for four NUE-related agronomic traits of the 225 rice varieties were separately analyzed (Table 1). The results showed that the correlations between the two nitrogen levels were different.

**TABLE 1:** Correlation analysis of the mean amount on low-nitrogen-induced growth response (LNGR) agronomic traits.

N level	Association between traits	Correlation	*P*-value
NN	SV * LL	–0.22	0.0008
	SV * PH	–0.49	8.07e-016
	SV * TN	0.20	0.002
	LL * PH	0.47	1.10e-014
	LL * TN	–0.26	3.69e-005
	PH * TN	–0.39	3.40e-010
LN	LSV * LLL	–0.15	0.026
	LSV * LPH	–0.28	0.00002
	LSV * LTN	0.03	0.667
	LLL * LPH	0.50	3.88e-016
	LLL * LTN	–0.19	0.004
	LPH * LTN	–0.43	7.03e-012

*NN, normal nitrogen; LN, low nitrogen; SV, soil and plant analyzer development value; LL, leaf length; PH, plant height; TN, tiller number.*

**FIGURE 2:**
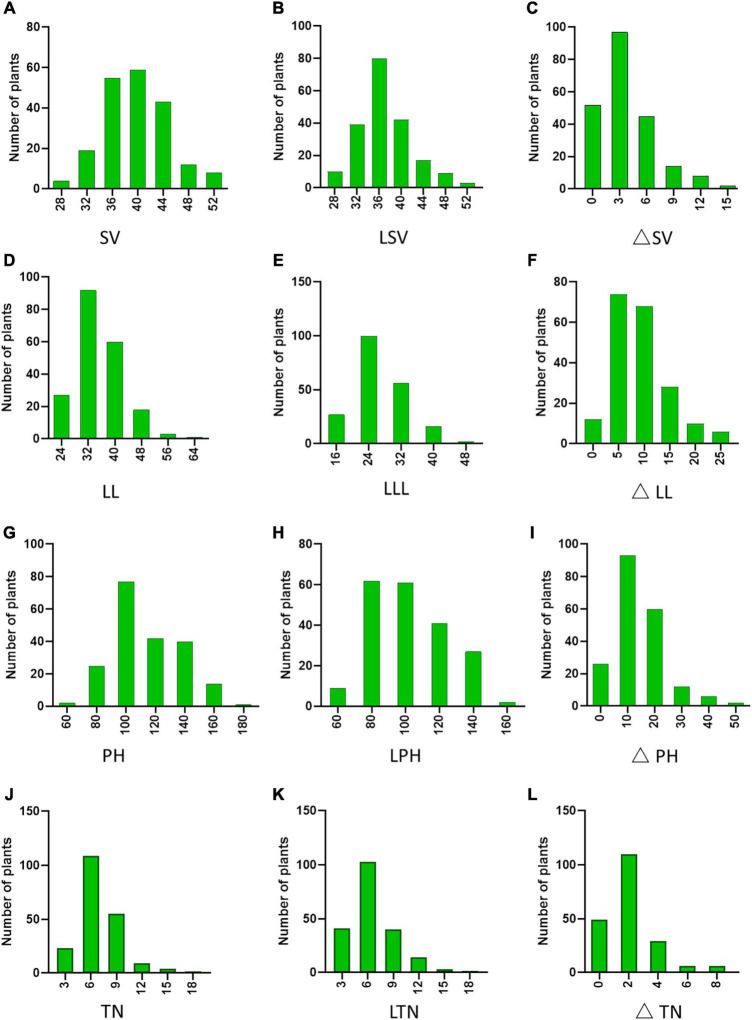
Phenotypic diversity of 225 rice accessions: **(A)** SV, **(D)** LL, **(G)** PH, and **(J)** TN at NN level. **(B)** SV, **(E)** LL, **(H)** PH, and **(K)** TN at LN level. Phenotypic difference (△NN-LN) of **(C)** SV, **(F)** LL, **(I)** PH, and **(L)** TN.

To confirm whether the phenotypic changes were related to nitrogen, we found that the mean values for the four traits under NN were higher than the mean values for the phenotypic data under LN (*P*_△ *SV*_, _△ *LL*_, _△ *PH*_ < 0.0001, *P*_△ *TN*_ < 0.05). The results were consistent with the frequency distribution of phenotypic data, and thus, the N treatment was proven to be effective (Figure 2). By contrast, not all phenotypic values were significantly reduced under LN conditions. To reflect LNGR, we focused on the difference (△NN-LN) between the phenotypic value of NN and LN (Table 2 and Supplementary Table 1). We divided the 225 rice varieties into hyposensitive and sensitive varieties with significantly different values. Notably, the phenotypic values of 45.16%, 13.82%, 23.04%, and 47.93% varieties were insignificantly reduced for SV at LN level (LSV), LL at LN level (LLL), PH at LN level (LPH), and TN at LN level (LTN), respectively, under LN. These varieties might be a target of selection for rice breeding and cultivation under LN conditions, especially Geng_7623 (HY124) and Huhui_91269 (HY206).

**TABLE 2:** Phenotypic differences in the four nitrogen-use efficiency (NUE)-related traits.

Trait	PV		Mean	SE	Max Value	Min Value	Skewness	Kurtosis	SD	CV (%)	PC (%)
	NN	LN									
**△**SV	39.84	37.04	3.60	0.30	15.18	0.09	0.35	0.51	4.42	19.49	45.16
**△**LL	34.62	26.49	7.98	0.40	27.07	0.67	0.51	0.90	6.09	37.04	13.82
**△**PH	111.49	99.18	12.65	0.72	50.10	0	0.24	1.59	10.93	19.48	23.04
**△**TN	7.03	5.83	2.08	0.17	8.83	0	–0.05	1.21	2.56	6.56	47.93

*PV, phenotypic value; CV, coefficient of variation; PC, phenotypic changes; NN, normal nitrogen; LN, low nitrogen; SV, soil and plant analyzer development value; LL, leaf length; PH, plant height; TN, tiller number.*

### Phylogenetic Analyses and Single-Nucleotide Polymorphisms

**FIGURE 3:**
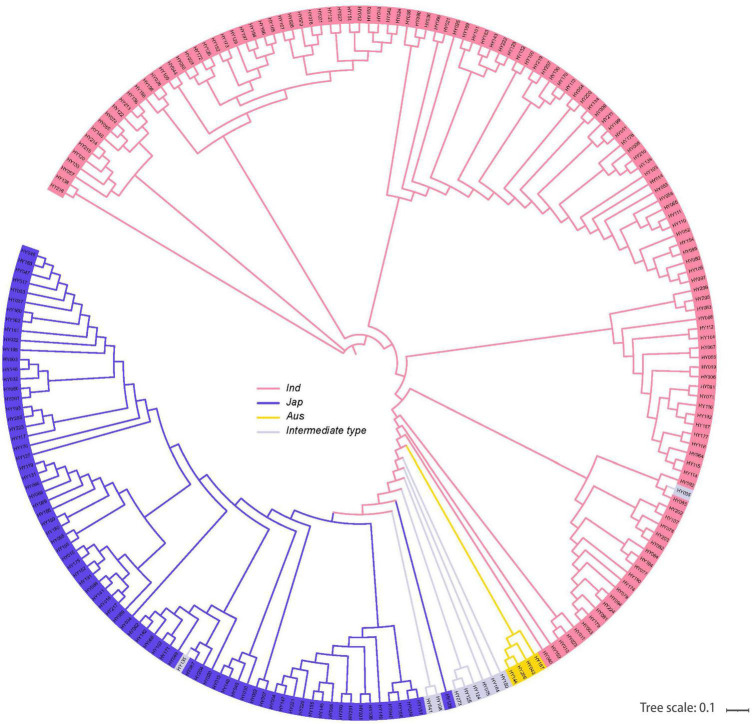
Phylogram of 225 natural varieties showing the divergence. Four main rice subpopulations indicated with different colors: *O. indica*, *O. japonica*, *Aus*, and *Intermediate types.*

We planted 225 natural rice varieties in NN (180 kg^⋅^hm^–2^) and LN (90 kg^⋅^hm^–2^) fields at CNRRI. Based on the genetic distance, we further identified neighbor-joining clustering and divided 225 varieties into 4 main rice subpopulations (Figure 3). The structural analysis showed that there are *indica*, *japonica*, *Aus*, and *Intermediate-type* varieties distributed in each subpopulation. The 225 varieties with NUE-related phenotypes originated from 5 geographic regions (Supplementary Figure 1). Varieties in Asia accounted for 85.71%, followed by America 4.15%, Europe 3.69%, Africa 3.69%, and Oceania 2.76% (Supplementary Figure 2).

### Genome-Wide Association Study and Candidate Analysis

**FIGURE 4:**
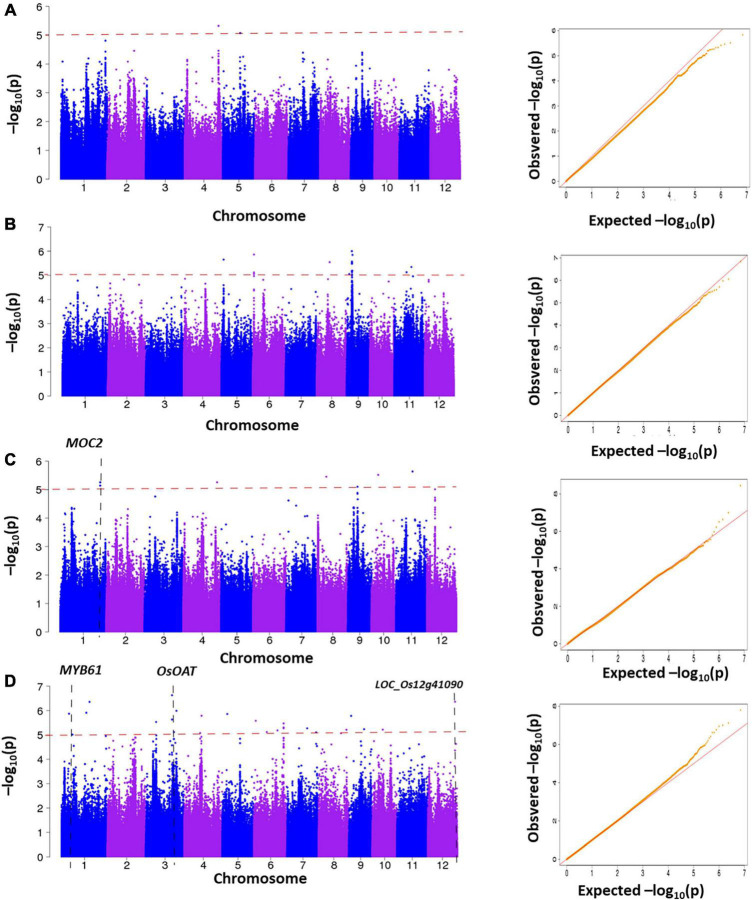
Manhattan plots generated from the genome-wide association study (GWAS) analysis were derived from the trait differences (△NN-LN) of NUE-related agronomic traits. **(A)** △SV, **(B)** △LL, **(C)** △PH, and **(D)** △TN.

We further performed GWAS on the difference of four LNGR agronomic traits under NN and LN levels to identify significantly associated genes (Figure 4). We selected the SNPs from the Rice 3K Sequencing Project that filtered a missing rate < 0.4 and a MAF > 0.05. We finally obtained 111,205 SNPs and performed an association analysis covering the entire genome with population structure corrections. It is generally considered that the SNP significant threshold is *P* ≤ 10^–5^, and linkage disequilibrium is 100 kb, as the literature queries showed (Lv et al., 2019).

**FIGURE 5:**
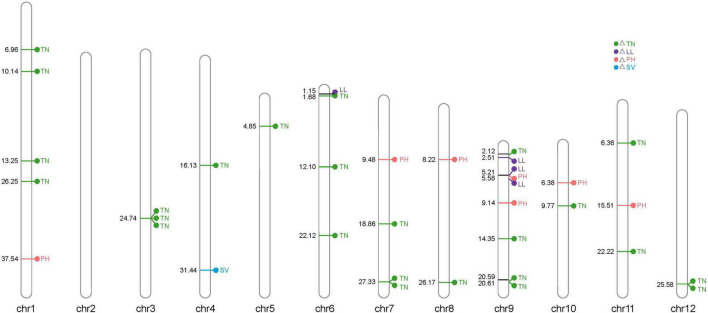
Distribution of 56 significantly associated single-nucleotide polymorphisms (SNPs) on 12 chromosomes based on the physical distance from the four NUE-related agronomic trait differences. The numbers on the left side of each column represent the physical location (Mb) of each lead SNP. Letters to the right of each column represent the corresponding traits of △SV, △LL, △PH, and △TN.

The genomic positions of known NUE-related functional genes with the GWAS intervals obtained in this study were compared. A total of 4 NUE-related candidate genes were detected after estimating 56 significantly associated SNPs distributed on the whole genome in 225 rice varieties. Notably, 2, 8, 20, and 26 significantly associated SNPs were obtained for △SV, △PH, △LL, and △TN, respectively, and each candidate region of the significant SNPs spanned 200 kb (i.e., 100 kb upstream and 100 kb downstream) (Figure 5 and Supplementary Table 2).

Using the previously reported genes and published studies regarding nitrogen as references, altogether, three LN-associated candidate genes corresponding to the significantly associated SNPs were commonly detected in our study (Table 3). In this study, *MYB61* was detected in the candidate interval of Chr01_10141302, *LOC_Os01g18240*, which is a well-known gene-modulating N use and C metabolism between rice *indica* and *japonica* subspecies. As a transcriptional factor, *MYB61* is directly regulated by a known NUE regulator, *GRF4*, which coordinates cellulosic biomass production and N utilization (Gao Y. et al., 2020).

**TABLE 3:** Summary of putative candidate genes for NUE.

Chr.	Gene ID	Gene name	SNP	Annotation
1	LOC_Os01g18240	*MYB61*	Chr01_10141302	MYB family transcription factor
1	LOC_Os01g64660	*MOC2*	Chr01_37543388	Monoculm 2; cytosolic fructose-1,6-bisphosphatase 1
3	LOC_Os03g44150	*OsOAT*	Chr03_24739537	Ornithine δ-aminotransferase
12	LOC_Os12g41090	Uncloned	Chr12_25582237	Calcium/calmodulin-dependent protein kinases

*SNP, single-nucleotide polymorphism.*

The candidate gene of *OsOAT*, *LOC_Os03g44150*, was located at the candidate interval of Chr03_24739537. *OsOAT* (i.e., ornithine δ-aminotransferase) plays a critical role in connecting arginine cycling and proline cycling and in contributing to floret development and seed setting in rice (Liu C. et al., 2018). On chromosome 1, we identified a candidate gene, *MOC2*, at the candidate interval of Chr01_37543388, whose mutants display significantly reduced TN, a reduced growth rate, and a consequent dwarf phenotype (Koumoto et al., 2013). We found that the typical N-related phenotypes changed, and therefore, we considered it to be a candidate gene for the subsequent haplotype analysis.

### Haplotype Analysis and Gene Mining

**FIGURE 6:**
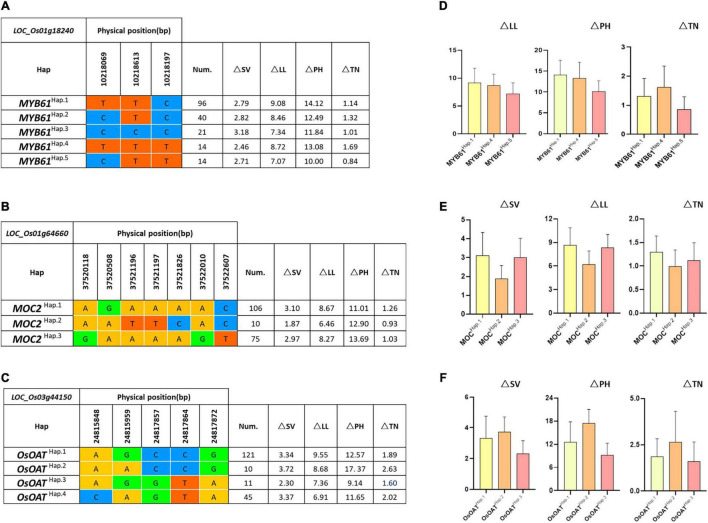
Haplotype analysis of known genes *MYB61*, *MOC2*, and *OsOAT*. Gene structures (left) and the mean amount on △SV, △LL, △PH, and △TN of different haplotypes (right) of **(A,D)** *MYB61* (*LOC_Os01g18240*), **(B,E)** *MOC2* (*LOC_Os01g64660*), and **(C,F)** *OsOAT* (*LOC_Os03g44150*). Num, number of accessions.

To further study the role of candidate genes, we conducted the haplotype analyses on the 225 rice varieties to identify the elite haplotypes. We first analyzed the sequences of *MYB61* in the 225 varieties. Based on the haplotype analysis and comparative analysis of the corresponding phenotypic data, all varieties possessed five types of haplotypes that play important roles in response to LN stress (Figures 6A,D). The varieties of *MYB61*^*Hap*^^⋅^^5^ contained CTT sequences, especially Menjiagao_1 (HY192) and Xiangaizao_10hao (HY197), and the phenotypic differences in the four traits were significantly or extremely significantly lower than others. We presumed that base differences affected the gene expression of *MYB61*, and *MYB61*^*Hap*^^⋅^^5^ performed more optimally in increasing the nitrogen utilization efficiency as an elite haplotype.

As the literature queries showed, *MOC2* is involved in N-related agronomic trait regulation. Importantly, *MOC2* has not been reported participating in N assimilation or utilization. The haplotype analysis (Figures 6B,E) indicates that out of 225 varieties, there are a total of 3 haplotypes at the *MOC2* locus. Accessions with *MOC2*^*Hap*^^⋅^^2^ showed minimal differences in SV, LL, and TN under NN or LN (i.e., △SV, △LL, and △TN). Thus, we speculated that *MOC2*^*Hap*^^⋅^^2^ may be associated with the NUE regulation.

Moreover, on chromosome 3, the candidate gene of *OsOAT* was predicted to encode ornithine δ-aminotransferase. Through published studies, we found that *OsOAT* regulates the N-related function of rice. Our results revealed that the accessions with *OsOAT*^*Hap*^^⋅^^3^ exhibit a lower difference of SV, PH, and TN (i.e., △SV, △PH, and △TN). The haplotype analysis suggests that *OsOAT*^*Hap*^^⋅^^3^ is strongly associated with NUE in rice (Figures 6C,F).

As expected, in addition to the published genes described in the sections earlier, several other genes encoding N-related protein were worthy of mining in our study. In this report, it was described that *OsCPK12* encodes calcium-dependent protein kinase and also participates in the signal transduction pathway in the LN stress response. Thus, it may be useful to include it when engineering crop plants with improved tolerance to LN levels (Takayuki et al., 2010). In our association panel, *LOC_Os12g41090* was located at the candidate interval of Chr12_25582237. It was annotated to associate with calcium/calmodulin-dependent protein kinases. Based on the gene annotation and the publicly available literature, we deduced that *LOC_Os12g41090* was a candidate gene involved in the NUE regulation.

## Discussion

Nitrogen (N) is an indispensable element for crop production. Excessive chemical nitrogen fertilizer is applied to obtain high crop yields (Krapp, 2015; Chen et al., 2016). The sequence-based GWAS is a highly efficient approach for analyzing rice agronomic traits, and it can identify the elite genes and can evaluate rice core accessions to assist in specifically improving the usage of nitrogen. In this study, we collected 225 worldwide rice germplasms and performed GWAS on the trait differences (△NN-LN) of NUE-related agronomic traits. More importantly, compared with the hydroponic or seedling stage phenotyping experiment, our study was completed in the field under two different N levels (NN, 180 kg^⋅^hm^–2^; LN, 90 kg^⋅^hm^–2^) until maturity. The results obtained exhibited meaning and persuasiveness.

Plant NUE is an inherently complex trait (Masclaux-Daubresse et al., 2010; Gao Z. et al., 2019; Gao H. et al., 2020). At present, some studies exist that focus on NUE in rice (Hu et al., 2015; Wang et al., 2018b; Zhang S. et al., 2021). However, the studies on NUE by the GWAS analysis are limited, and to the best of our knowledge, few studies have been previously performed. In rice, a NIN-like protein, *OsNLP4*, was associated with NUE and was identified through GWAS in rice core populations. The previous studies showed that *OsNLP4* imparts nitrogen assimilation efficiency and promotes the number of tillers and yield of rice (Yu et al., 2021). Additionally, *OsNPF6.1*^*HapB*^ is an elite haplotype derived from the GWAS analysis of extreme nitrogen-related phenotypes, such as PH, effective panicle number (EPN), and yield per plant (YPP) (Tang et al., 2019).

We measured the LN-related agronomic traits of SV, LL, PH, and TN of the 225 rice varieties under a NN level and an LN level. There was a correlation between different LN-related traits under NN and LN levels, suggesting that there were close physiological bases among different traits (Xu et al., 2012). We, therefore, focused on the difference (△NN-LN) between the phenotypic value of NN and LN and divided the varieties into hyposensitive and sensitive varieties. The critical first step before initiating association mapping is to collect a wide range of germplasm resources with different genetic backgrounds (Zheng M. et al., 2017). Our selection of 225 rice germplasms covered 5 continents around the world, which indicated that the results for genetic diversity were reliable for a feasible GWAS. Furthermore, we selected high-quality SNPs from the Rice 3K Sequencing Project and performed the association analysis on the phenotypic value difference (△NN-LN) covering the entire genome.

Numerous studies have supported the idea that N regulation is involved in plant development (Zhang S. et al., 2020; Zhang M. et al., 2021). Based on the previously reported genes and published studies, we conducted candidate gene identification. More importantly, the reliability of our GWAS for association mapping of NUE was previously confirmed by Gao (Gao Y. et al., 2020), who found that the *indica* allele of *MYB61* exhibited superiority in governing nitrogen use and biomass production. Encouragingly, we also identified the NUE-associated genes *OsOAT* and *MOC2* from our GWAS, and both of them play a critical role in modulating N use. Additionally, we also identified several other genes encoding N-related proteins worthy of mining in our study.

As expected, the gene *LOC_Os12g41090*, which encodes calcium/calmodulin-dependent protein kinases, displays a signal close to *OsCPK12*. *OsCPK12* itself can participate in the signal transduction pathway in the LN stress response and can potentially improve the NUE of rice. One concern with studying this candidate gene is that the gene function must be verified. We, therefore, have constructed various vectors for transgenic verification. There are multiple haplotypes of genes in different varieties, which cause differences in phenotypic value. *MYB61* was classified into five haplotypes based on three missense SNPs in its coding region. *MYB61*^*Hap*^^⋅^^5^ was found to be an elite haplotype for its lower phenotypic difference (△NN-LN). *MOC2* regulates the N-related function of rice, and further haplotype analysis revealed that *MOC2* ^*Hap*^^⋅^^2^ is the elite haplotype that enhances NUE. A previous study suggested that *OsOAT* has an important role in regulating NUE, and *OsOAT*^*Hap*^^⋅^^3^ was significantly associated with the NUE of rice compared with other haplotypes. Moreover, Menjiagao_1 and Xiangaizao_10hao with *MYB61*^*Hap*^^⋅^^5^ were identified as promising candidate rice accessions in NUE under LN-induced phenotypic data, which have shown an insignificant phenotypic difference (△NN-LN).

Different varieties responded differently to N levels, and the physiological differences may be caused by genotype and N interactions (Watanabe and Inubushl, 1986; He et al., 2020). The analysis of agronomic traits for two different N levels suggests a critical role for NUE. We successfully deduced that four candidate genes are involved in N regulation, and these were consistent with what currently appears in the available literature. Importantly, our research provides a theoretical basis for the selection and breeding of NUE genes and materials.

## Data Availability Statement

The original contributions presented in the study are included in the article/Supplementary Material, further inquiries can be directed to the corresponding author/s.

## Author Contributions

YL, LG, QQ, and LS conceived and designed the study. LS and QW performed GWAS. YW and XL provided advice for the experimental design. YL, JM, HH, and YW performed the phenotyping measurement. YL, JM, and YW performed the data analysis. YL wrote the manuscript. All authors have read the manuscript and approved it for publication.

## Conflict of Interest

The authors declare that the research was conducted in the absence of any commercial or financial relationships that could be construed as a potential conflict of interest.

## Publisher’s Note

All claims expressed in this article are solely those of the authors and do not necessarily represent those of their affiliated organizations, or those of the publisher, the editors and the reviewers. Any product that may be evaluated in this article, or claim that may be made by its manufacturer, is not guaranteed or endorsed by the publisher.

## Footnotes

1
https://snp-seek.irri.org/

2
http://www.ricedata.cn/gene/
