# Supplementary material for: Loci and Natural Alleles for Low-Nitrogen-Induced Growth Response Revealed by the Genome-Wide Association Study Analysis in Rice (Oryza sativa L.)
Source: Front Plant Sci. 2021 Nov 5;12:770736. doi: 10.3389/fpls.2021.770736 (PMC8602835; doi:10.3389/fpls.2021.770736)
Supplement: Supplementary file 1 [file Data_Sheet_1.docx]

Supplementary Material


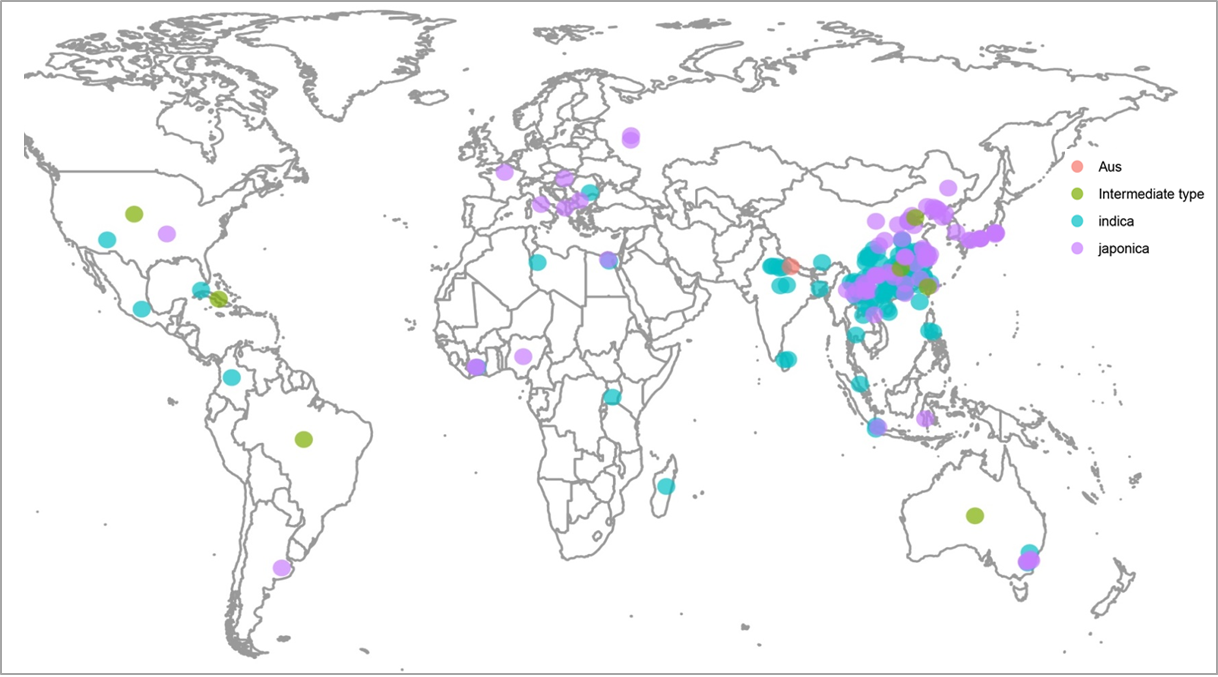


**Supplementary FIGURE S1**| Location of collected natural varieties from the worldwide. The circle color represents the subpopulations of varieties.


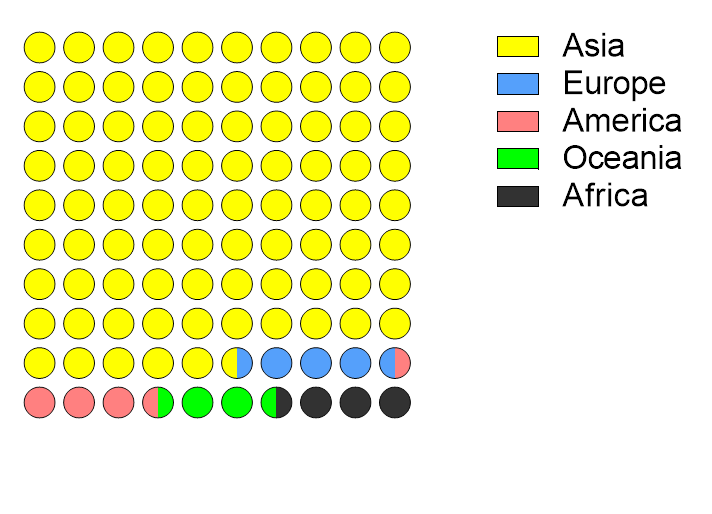


**Supplementary FIGURE S2**| Geographical distribution percentage of 225 natural varieties on five continents.The pie chart size and color is proportional to the percentage of accessions.

**Supplementary Table S1 | Summary of 225 rice natural varieties, origin and phenotypic differences value**

| Num. | Subpopulation | Ori. | Varname | △SV | △LL(cm) | △PH(cm) | △TN |  |  |  |
| --- | --- | --- | --- | --- | --- | --- | --- | --- | --- | --- |
| HY001 | Japonica | China | Heibiao | 6.43 | 3.84 | 1.97 | 5.00 |  |  |  |
| HY002 | Japonica | China | Sansuijin | 7.18 | 3.87 | 12.34 | 0.33 |  |  |  |
| HY003 | Japonica | China | Zaoshengbai__ | 0.76 | 4.57 | 24.33 | 4.80 |  |  |  |
| HY004 | Japonica | Japan | Qiuguangtengxi_104_ | - | 5.55 | 14.00 | 5.07 |  |  |  |
| HY005 | Japonica | Japan | Wanshi | 1.16 | 5.38 | 10.33 | 0.77 |  |  |  |
| HY006 | Indica | Vietnam | Yikong | 0.78 | 2.01 | 11.67 | 1.27 |  |  |  |
| HY007 | Japonica | Vietnam | Baxiang_ | 3.07 | 9.48 | 30.33 | 0.85 |  |  |  |
| HY008 | Indica | Vietnam | Vietnam_Zaodao | 2.88 | 4.83 | 0.75 | 0.50 |  |  |  |
| HY009 | Indica | Malaysia | Malaihong_ | 2.92 | 9.17 | 0.84 | 4.00 |  |  |  |
| HY010 | Indica | India | CO_22 | 5.10 | 23.35 | 6.34 | 3.20 |  |  |  |
| HY011 | Indica | India | 2037(Rajahamsal) | 6.65 | 24.30 | 12.50 | 0.13 |  |  |  |
| HY012 | Indica | Sri lanka | Sri_Lanka_1_ | 7.00 | 10.93 | 15.17 | 2.10 |  |  |  |
| HY013 | Japonica | Soviet Union | Wuziluosi_215 | 15.18 | 17.66 | 12.00 | 1.40 |  |  |  |
| HY014 | Indica | Romania | Kahamu | 3.48 | 9.67 | 2.33 | 1.33 |  |  |  |
| HY015 | Japonica | Hungary | Aomierte_168 | 3.26 | 11.13 | 11.33 | 2.00 |  |  |  |
| HY016 | Japonica | Bulgaria | Aerjituo | 3.59 | 10.77 | 2.50 | 7.17 |  |  |  |
| HY017 | Japonica | United States | American_Huangkedao | 5.26 | 4.43 | 13.16 | 2.66 |  |  |  |
| HY018 | Indica | Mexico | Buleida_A-75 | 1.22 | 2.88 | 9.83 | 0.10 |  |  |  |
| HY019 | Indica | Australia | Seln_244A6-20 | 1.65 | 2.47 | 12.50 | 3.87 |  |  |  |
| HY020 | Japonica | North Korea | Gaoliqiu | 1.00 | 1.00 | - | - |  |  |  |
| HY021 | Indica | Thailand | Xianluosichi | 1.06 | 15.23 | 8.83 | 2.47 |  |  |  |
| HY022 | Japonica | Indonesia | Djanda_Mandja | 5.70 | 3.97 | 23.67 | 0.66 |  |  |  |
| HY023 | Indica | Indonesia | Padi Ladang Ase Polo Komek | 1.00 | - | - | - |  |  |  |
| HY024 | Indica | Phillipines | C_894-21 | 4.03 | 7.60 | 17.33 | 0.83 |  |  |  |
| HY025 | Indica | Phillipines | IR_10179-23-1-3 | - | 9.25 | 7.50 | 2.00 |  |  |  |
| HY026 | Indica | India | Dumai | 2.24 | 9.83 | 28.93 | 2.25 |  |  |  |
| HY027 | Indica | India | Jaibattey | 3.40 | 3.30 | 19.53 | 1.00 |  |  |  |
| HY028 | Indica | India | Rohini | 2.02 | 0.67 | 12.17 | 1.83 |  |  |  |
| HY029 | Indica | Sri lanka | BW_293-2 | 6.23 | 7.05 | 9.00 | 2.66 |  |  |  |
| HY030 | Japonica | Albania | Albania_Rice | 6.99 | 14.46 | 13.17 | 2.97 |  |  |  |
| HY031 | Indica | United States | American Rice | 1.00 | - | - | - |  |  |  |
| HY032 | Japonica | Brazil | Jijucas Claro | 1.00 | - | - | - |  |  |  |
| HY033 | Japonica | Argentina | Nanoay_P.A | 1.95 | 8.09 | 26.50 | 0.50 |  |  |  |
| HY034 | Japonica | Egypt | Nabated_A_Smar | 3.41 | 16.68 | 16.00 | 0.83 |  |  |  |
| HY035 | Indica | Ivory Coast | IRAT_10 | 9.83 | 17.12 | 14.08 | 1.66 |  |  |  |
| HY036 | Indica | Uganda | K_24 | 13.61 | 27.07 | 7.00 | 1.00 |  |  |  |
| HY037 | Japonica | Australia | YR 83-23-11 | 1.00 | - | - | - |  |  |  |
| HY038 | Japonica | China | Qianchonglang_2 | 9.23 | 10.83 | 16.00 | 0.16 |  |  |  |
| HY039 | Japonica | Japan | Gongchengxiang_ | 7.69 | 7.38 | 14.17 | 1.00 |  |  |  |
| HY040 | Japonica | Japan | Qiutianxiaoting | 7.70 | 4.44 | 19.00 | 3.17 |  |  |  |
| HY041 | admix | Japan | Zhenfu_8 | 6.84 | 5.33 | 24.17 | 1.33 |  |  |  |
| HY042 | Indica | Indonesia | CISOKAN | 7.35 | 7.35 | 19.50 | 7.40 |  |  |  |
| HY043 | Aus | Nepal | Zacaodao 13 | 1.00 | - | - | - |  |  |  |
| HY044 | Indica | Egypt | GZ_1368-5-4 | 4.59 | 4.42 | 19.50 | 5.25 |  |  |  |
| HY045 | Indica | Madagascar | J34 | 2.02 | 7.04 | 33.08 | 2.00 |  |  |  |
| HY046 | Japonica | Australia | 80A97YR303-304-1-3 | 6.49 | 3.15 | 22.10 | 1.84 |  |  |  |
| HY047 | Japonica | Australia | 80050YR72136-43 | 4.10 | 9.02 | 9.97 | 2.50 |  |  |  |
| HY048 | Japonica | Australia | YR196 | 3.83 | 12.93 | 1.33 | 0.17 |  |  |  |
| HY049 | Japonica | China | Xiushui_115 | 3.51 | 5.69 | 14.67 | - |  |  |  |
| HY050 | Indica | China | Erjiunan_1 | 6.12 | 10.75 | 11.17 | 2.17 |  |  |  |
| HY051 | Indica | China | Nanjing_11 | 12.18 | 5.22 | - | 2.60 |  |  |  |
| HY052 | Indica | China | Aijiaonante | 11.21 | 11.48 | 10.17 | 1.60 |  |  |  |
| HY053 | Indica | China | Guangluai_4 | 7.83 | 6.03 | 17.13 | 1.17 |  |  |  |
| HY054 | Indica | China | Nantehao_ | 2.47 | 10.03 | 40.33 | 0.84 |  |  |  |
| HY055 | Indica | China | Guizhao_2 | 1.00 | - | 20.67 | 1.50 |  |  |  |
| HY056 | Indica | China | Xiangzaoxian_7 | - | 10.70 | 10.00 | 2.84 |  |  |  |
| HY057 | Indica | China | Huangsiguizhan | 0.42 | 8.12 | 21.17 | 2.66 |  |  |  |
| HY058 | Indica | China | Funingzipigengzi | 6.94 | 16.83 | 10.00 | 1.67 |  |  |  |
| HY059 | Japonica | China | Gaoyangdiandao_dahongmang | 11.48 | 20.31 | 20.83 | 0.50 |  |  |  |
| HY060 | Japonica | China | Dandongludao_ | 5.09 | 8.10 | 11.50 | 0.67 |  |  |  |
| HY061 | Japonica | China | Laoguangtou_83 | 9.02 | 9.40 | 13.00 | 0.83 |  |  |  |
| HY062 | Japonica | China | Muxiqiu | 1.98 | 14.84 | 20.17 | 3.30 |  |  |  |
| HY063 | Indica | China | Qiuqianbai_ | 1.87 | 15.55 | 10.33 | 1.84 |  |  |  |
| HY064 | Indica | China | Jinxibai2_ | 2.50 | 19.59 | 19.17 | 2.66 |  |  |  |
| HY065 | Indica | China | Taishannuo | 9.13 | 17.62 | 1.84 | 2.50 |  |  |  |
| HY066 | Indica | China | Jinbaoyin_ | 4.56 | 11.14 | 5.83 | 2.33 |  |  |  |
| HY067 | Indica | China | Minbeiwanxian | 2.88 | 8.18 | 19.67 | 0.20 |  |  |  |
| HY068 | Japonica | China | Yizhixiang | 4.16 | 7.45 | 8.33 | 1.83 |  |  |  |
| HY069 | Indica | China | Esiniu_ | 0.27 | 14.47 | 10.00 | 0.67 |  |  |  |
| HY070 | Indica | China | Heidu_4 | 4.53 | 7.80 | 10.50 | 3.17 |  |  |  |
| HY071 | Indica | China | Qiyuexian | 1.00 | - | - | - |  |  |  |
| HY072 | Japonica | China | Wuyunjing_7 | 8.67 | 6.82 | 15.67 | 2.00 |  |  |  |
| HY073 | admix | China | Bawangbian_1 | 2.45 | 10.18 | 4.00 | 1.33 |  |  |  |
| HY074 | Indica | China | Xugunuo | 1.56 | 9.27 | 6.50 | 1.67 |  |  |  |
| HY075 | admix | China | Muguanuo_ | 0.05 | 15.61 | 22.83 | 1.33 |  |  |  |
| HY076 | Indica | China | SanQishiluo | 8.12 | 22.20 | 5.00 | 1.83 |  |  |  |
| HY077 | Indica | China | Qitoubaigu3 | 5.49 | 23.97 | 26.66 | 2.00 |  |  |  |
| HY078 | Indica | China | Zimi | 3.92 | - | 5.90 | 3.16 |  |  |  |
| HY079 | Indica | China | Xiaohonggu | 3.28 | 10.97 | 15.90 | 0.83 |  |  |  |
| HY080 | Indica | China | Gongju_73 | 1.77 | 11.45 | 15.50 | 1.67 |  |  |  |
| HY081 | Indica | China | Qitougu | 1.75 | 7.15 | 32.83 | 1.67 |  |  |  |
| HY082 | Indica | China | Zinuo | 3.20 | 15.23 | 36.33 | 1.33 |  |  |  |
| HY083 | Indica | China | Mowangguneiza | 3.32 | 8.27 | 44.66 | 2.16 |  |  |  |
| HY084 | Indica | China | Jinzhinuo4_ | 0.72 | 11.69 | 10.66 | 1.30 |  |  |  |
| HY085 | Indica | China | Jiienuo | 1.21 | 22.07 | 9.33 | 1.00 |  |  |  |
| HY086 | Japonica | China | Hongkezhenuo_ | 2.77 | 25.55 | 42.50 | 1.67 |  |  |  |
| HY087 | Japonica | China | Yangkenuo | 0.33 | 10.83 | 17.83 | 2.83 |  |  |  |
| HY088 | Japonica | China | Maguzi_ | 5.01 | 15.78 | 16.03 | 1.97 |  |  |  |
| HY089 | Japonica | China | Laohongdao | 3.96 | 11.88 | 8.17 | 0.50 |  |  |  |
| HY090 | Indica | India | Jiabala | 1.91 | 3.87 | 4.50 | 3.00 |  |  |  |
| HY091 | Indica | China | Baoxuan_21 | - | 13.33 | 23.50 | 1.33 |  |  |  |
| HY092 | Indica | China | Wenxiangnuo | 2.61 | 4.80 | 18.17 | 3.67 |  |  |  |
| HY093 | Indica | China | Teqing | 2.31 | 6.02 | 9.84 | 0.50 |  |  |  |
| HY094 | Indica | China | Xianggu | 1.73 | 11.73 | 15.83 | 2.50 |  |  |  |
| HY095 | Japonica | China | Lengshuigu_2 | 1.28 | 2.26 | 30.50 | 2.17 |  |  |  |
| HY096 | Japonica | China | Zimangfeie_ | 3.87 | 8.69 | 27.00 | 0.33 |  |  |  |
| HY097 | Indica | China | Liusha_1 | 2.17 | 8.77 | 10.34 | 2.00 |  |  |  |
| HY098 | Indica | China | Chenwan_3_ | 3.09 | 16.49 | 15.50 | 4.73 |  |  |  |
| HY099 | Indica | China | Chengduai_3_ | 5.14 | 13.96 | 12.33 | 0.00 |  |  |  |
| HY100 | Indica | China | Aimakang | 0.38 | 6.58 | 13.00 | 0.33 |  |  |  |
| HY101 | Indica | China | Shufeng_101 | 4.32 | 6.97 | 9.17 | 3.17 |  |  |  |
| HY102 | Japonica | China | Lixingeng | 1.00 | - | - | - |  |  |  |
| HY103 | Indica | China | Guangluai_15-1 | 2.85 | 9.25 | 12.83 | 1.17 |  |  |  |
| HY104 | Indica | China | Hongwan_1_ | 8.76 | 14.93 | 15.00 | 2.67 |  |  |  |
| HY105 | Indica | China | Luke_3_ | 4.61 | 6.56 | 16.67 | 1.07 |  |  |  |
| HY106 | Japonica | China | Liaogeng_287 | 1.02 | 7.03 | 7.66 | 0.33 |  |  |  |
| HY107 | Indica | China | Zaoshuxiangheimi | 2.02 | 8.32 | 22.00 | 0.20 |  |  |  |
| HY108 | admix | China | Geng_87-304 | 1.78 | 5.02 | 15.67 | 1.07 |  |  |  |
| HY109 | Indica | China | Xiangwanxian_3__ | 10.39 | 7.75 | 7.84 | 1.33 |  |  |  |
| HY110 | Indica | China | Zaoxian_240 | 13.20 | 5.03 | 9.67 | 1.66 |  |  |  |
| HY111 | Indica | China | Dangyu_5_ | 0.96 | 3.97 | 9.33 | 4.20 |  |  |  |
| HY112 | Indica | China | Hongainuo | 1.10 | 1.00 | 22.67 | 1.33 |  |  |  |
| HY113 | Indica | China | Wanlixian | 1.75 | 11.82 | 17.66 | 1.34 |  |  |  |
| HY114 | Indica | China | Aizizhan | 0.20 | 4.87 | 12.33 | 4.83 |  |  |  |
| HY115 | Indica | China | Xiaobaimi | 2.13 | 4.56 | 2.00 | - |  |  |  |
| HY116 | Indica | China | Yanshuichi | 6.24 | 13.52 | 9.00 | 8.83 |  |  |  |
| HY117 | Japonica | China | Xishi_15 | 3.49 | 12.65 | 25.33 | 0.07 |  |  |  |
| HY118 | Indica | China | Honggenghangu3_ | 3.03 | 4.31 | 22.33 | 0.34 |  |  |  |
| HY119 | Japonica | China | Yuyannuo | 2.10 | 9.15 | 28.50 | 1.50 |  |  |  |
| HY120 | Indica | Phillipines | 80B | 3.05 | 5.07 | 9.17 | 2.50 |  |  |  |
| HY121 | Indica | China | Gu_154 | 2.08 | 6.57 | 15.00 | 1.33 |  |  |  |
| HY122 | Indica | China | IR_661-1 | 1.99 | 10.80 | 9.50 | 1.16 |  |  |  |
| HY123 | Indica | China | Pei_C122 | 2.65 | 12.42 | 15.16 | 1.87 |  |  |  |
| HY124 | admix | China | Geng_7623 | 0.89 | 2.02 | 7.17 | 0.33 |  |  |  |
| HY125 | admix | China | Ninghui_21 | 3.83 | 6.10 | 6.17 | 0.16 |  |  |  |
| HY126 | Japonica | China | 76--1 | 1.35 | - | - | - |  |  |  |
| HY127 | Japonica | China | Huhui 628 | 1.50 | - | - | - |  |  |  |
| HY128 | Indica | China | Teqingxuanhui | 0.47 | 2.77 | 4.00 | 2.60 |  |  |  |
| HY129 | Indica | China | JWR_221 | 2.49 | 7.42 | 13.17 | 0.17 |  |  |  |
| HY130 | Indica | China | Baikehanhe | 4.24 | 10.04 | 14.50 | 2.90 |  |  |  |
| HY131 | Japonica | China | Lengshuinuo | 2.23 | 10.55 | 19.17 | 1.60 |  |  |  |
| HY132 | Indica | China | Haoxiang_ | 1.01 | 4.44 | 21.33 | 0.17 |  |  |  |
| HY133 | Indica | China | L_301B | 3.74 | 19.62 | 24.00 | 1.53 |  |  |  |
| HY134 | Indica | China | Jinnante_43B | 7.16 | 16.68 | 16.75 | 1.13 |  |  |  |
| HY135 | Japonica | China | Zaoshunonghu_6 | 12.36 | 14.35 | 28.50 | 3.50 |  |  |  |
| HY136 | Indica | China | Qingsiai_16B | 1.35 | 7.82 | 8.17 | 3.00 |  |  |  |
| HY137 | GJ-tmp | China | Liming_B | - | 7.84 | 13.83 | 1.33 |  |  |  |
| HY138 | Indica | China | Baoxie-7B | 1.12 | 19.43 | 19.66 | 4.33 |  |  |  |
| HY139 | Indica | China | G_Zhenshan_97B | 5.19 | 10.75 | 15.83 | 1.00 |  |  |  |
| HY140 | Indica | China | Taizhongzailai_1/taizhong65 | 2.17 | 12.47 | 16.13 | 2.33 |  |  |  |
| HY141 | Japonica | China | Yelicanghua | 7.34 | 20.68 | 17.83 | 3.16 |  |  |  |
| HY142 | Japonica | China | Baigedao | 2.31 | 7.33 | 15.66 | 0.30 |  |  |  |
| HY143 | Indica | China | Liushizao_ | 3.09 | 2.67 | 0.17 | 0.40 |  |  |  |
| HY144 | Aus | China | Qingke | 5.43 | 4.85 | 4.34 | 2.07 |  |  |  |
| HY145 | Japonica | North Korea | Jianghuadao_ | 0.25 | 15.52 | 6.34 | 2.83 |  |  |  |
| HY146 | Japonica | Japan | Zhuyuan | 0.15 | 6.22 | 2.17 | 1.17 |  |  |  |
| HY147 | Japonica | Japan | Ailuyu | 0.09 | 5.89 | 19.50 | - |  |  |  |
| HY148 | Japonica | Russia | Hongse_90 | 9.32 | 5.28 | 18.00 | 1.50 |  |  |  |
| HY149 | Japonica | Italy | Linguo | 6.12 | 9.50 | 18.83 | 1.80 |  |  |  |
| HY150 | admix | Indonesia | Tjantajan | 0.15 | 7.92 | 9.33 | 0.50 |  |  |  |
| HY151 | Indica | Bangladesh | BRC_25-146-2-1 | 3.29 | 3.88 | 8.83 | 1.00 |  |  |  |
| HY152 | Japonica | France | Keluoduo_B | 2.72 | 12.15 | 13.16 | 2.77 |  |  |  |
| HY153 | Indica | Columbia | P1790-5-1M-4-5M-1B-3M-B | 4.26 | 3.77 | 17.84 | 1.17 |  |  |  |
| HY154 | Indica | Australia | 71011 | 5.10 | 0.68 | 11.00 | 2.34 |  |  |  |
| HY155 | Japonica | Japan | Chimao | 11.64 | 4.35 | 6.84 | 0.50 |  |  |  |
| HY156 | Japonica | Japan | Qingnuo_Kyohatamochi | 0.40 | 14.10 | 0.00 | 1.00 |  |  |  |
| HY157 | Indica | Vietnam | CHANH_148 | 3.23 | 11.76 | 11.83 | 3.00 |  |  |  |
| HY158 | Indica | Laos | SLK_2-18-2 | 6.13 | 11.68 | 10.17 | 2.00 |  |  |  |
| HY159 | Indica | India | RP_1570-44-1 | 5.77 | 2.93 | 0.34 | 8.50 |  |  |  |
| HY160 | Japonica | Ivory Coast | IRAT_36 | 0.85 | 14.86 | 20.83 | 0.16 |  |  |  |
| HY161 | Japonica | Ivory Coast | IRAT_669 | 3.10 | 8.69 | 2.67 | 0.50 |  |  |  |
| HY162 | Japonica | Nigeria | ITA_221 | 0.65 | 3.43 | 8.66 | 1.17 |  |  |  |
| HY163 | Japonica | Australia | 80A60YR71009-1-5 | 6.53 | 12.72 | 24.17 | 3.50 |  |  |  |
| HY164 | admix | Phillipines | R42 | 8.09 | 1.88 | 9.83 | 1.00 |  |  |  |
| HY165 | Indica | Cuba | C.MEDIO 7 | 2.72 | - | - | - |  |  |  |
| HY166 | Indica | Cuba | ECIA_179-S13 | 5.55 | 9.16 | 11.17 | 1.50 |  |  |  |
| HY167 | Indica | India | PMS_10B | 7.95 | 5.98 | 10.50 | 1.33 |  |  |  |
| HY168 | Japonica | China | Taidongludao_ | 5.91 | 9.59 | 15.33 | 2.50 |  |  |  |
| HY169 | Indica | China | Taizhongxianxuan 2 | 0.15 | - | - | - |  |  |  |
| HY170 | Indica | China | Jiefangxian_ | 2.33 | 8.90 | 0.67 | 0.00 |  |  |  |
| HY171 | Japonica | China | Hongmisandan | 2.50 | 4.76 | 3.33 | 1.67 |  |  |  |
| HY172 | Indica | China | Jinyou 1 | - | - | 5.00 | 1.00 |  |  |  |
| HY173 | Indica | China | Chengnongshuijing_ | 0.45 | 3.65 | 8.17 | 1.73 |  |  |  |
| HY174 | Indica | China | Biwusheng_ | 5.08 | 8.08 | 34.00 | 0.16 |  |  |  |
| HY175 | Japonica | China | Longhuamaohu | 3.49 | 6.67 | 23.83 | 2.17 |  |  |  |
| HY176 | Japonica | China | Cunsanli | 2.37 | 7.30 | 11.34 | 0.34 |  |  |  |
| HY177 | Indica | China | Aihechi | 5.56 | 14.18 | 1.00 | 3.77 |  |  |  |
| HY178 | Indica | China | Lucaihao_ | 1.11 | 3.32 | 15.50 | 1.67 |  |  |  |
| HY179 | Indica | China | Nanxiongzaoyouzhan | 4.04 | 12.92 | 50.10 | 7.53 |  |  |  |
| HY180 | Japonica | China | Chikenuo | 12.66 | 0.96 | 11.66 | 3.75 |  |  |  |
| HY181 | Indica | China | Nanjing_6 | 4.64 | 14.15 | 16.00 | 0.50 |  |  |  |
| HY182 | Indica | China | Xuanenchangtanqingzhan | 0.78 | 10.28 | 5.50 | 0.67 |  |  |  |
| HY183 | Indica | China | Hanmadao4_ | 10.61 | 8.20 | 6.83 | 4.17 |  |  |  |
| HY184 | Indica | China | Honggu | 3.09 | 7.13 | 0.17 | 1.33 |  |  |  |
| HY185 | Japonica | China | Wuzidui | 1.84 | 10.48 | 36.84 | 2.66 |  |  |  |
| HY186 | Indica | Phillipines | IR24 | 5.50 | 3.02 | 15.34 | 3.00 |  |  |  |
| HY187 | Aus | China | Fanhaopi_ | 0.76 | 20.01 | 12.00 | 2.66 |  |  |  |
| HY188 | Indica | China | Shuhui498 | 3.78 | 7.95 | 18.16 | 0.00 |  |  |  |
| HY189 | Japonica | China | Cungunuo | 1.82 | 3.08 | 15.25 | 1.58 |  |  |  |
| HY190 | Indica | China | Younian | 4.07 | 10.26 | 23.83 | 0.66 |  |  |  |
| HY191 | Japonica | China | Heimangdao | 2.34 | 11.64 | 20.66 | 0.50 |  |  |  |
| HY192 | Japonica | China | Menjiagao_1 | 1.63 | 15.14 | 3.50 | 5.43 |  |  |  |
| HY193 | Japonica | China | Haobayong_1 | 3.04 | 7.26 | 37.33 | 3.33 |  |  |  |
| HY194 | Indica | China | Menjiading_2 | 3.17 | 5.91 | 12.17 | 3.84 |  |  |  |
| HY195 | Japonica | China | Banjiemang2 | 2.00 | - | - | - |  |  |  |
| HY196 | Indica | China | None | 0.24 | 0.70 | 10.50 | 1.20 |  |  |  |
| HY197 | Indica | China | Xiangaizao_10hao | 1.49 | 7.87 | 7.00 | 1.50 |  |  |  |
| HY198 | Indica | China | Xiangwanxian_1__ | 3.20 | 5.68 | 2.00 | 0.17 |  |  |  |
| HY199 | Indica | China | Aituogu_151 | 0.51 | 3.23 | 10.50 | 1.67 |  |  |  |
| HY200 | Japonica | China | Zhonghua_8 | 4.49 | 7.62 | 8.00 | 3.87 |  |  |  |
| HY201 | Japonica | China | Jindao_1 | 4.80 | 10.36 | 5.50 | 6.17 |  |  |  |
| HY202 | Indica | China | Momi | 3.33 | 5.84 | 11.84 | 1.67 |  |  |  |
| HY203 | Indica | China | Zhendao_232 | 1.16 | 4.38 | 5.00 | 1.50 |  |  |  |
| HY204 | Japonica | China | Zhengdao_5 | 6.90 | 11.30 | 12.67 | 0.93 |  |  |  |
| HY205 | Japonica | China | Lamujia | 3.46 | 5.23 | - | 6.85 |  |  |  |
| HY206 | Aus | China | Huhui_91269 | 1.55 | 1.19 | 1.67 | 3.97 |  |  |  |
| HY207 | Indica | China | Xiangdao_ | 2.26 | 6.07 | 16.83 | 3.87 |  |  |  |
| HY208 | Indica | China | Laozaogu | 3.23 | 1.49 | 7.34 | 1.84 |  |  |  |
| HY209 | Indica | China | Jinnante_B | 7.33 | 10.45 | 7.33 | 2.34 |  |  |  |
| HY210 | Indica | China | Zhuzhen_B | 4.02 | 6.27 | 3.84 | 0.83 |  |  |  |
| HY211 | Indica | China | Chaoyangyihao_B | 6.21 | 6.35 | 6.84 | 0.50 |  |  |  |
| HY212 | Indica | United States | lemont | 3.78 | 1.38 | 4.83 | 1.23 |  |  |  |
| HY213 | Indica | China | Xiangai_B | 0.59 | 8.37 | 12.16 | 1.16 |  |  |  |
| HY214 | Indica | China | Jiangnongzao_1_B | 5.65 | 6.98 | 4.17 | 3.83 |  |  |  |
| HY215 | Indica | China | Jinghu3_B | 2.47 | 7.27 | 9.00 | 2.34 |  |  |  |
| HY216 | Indica | China | Dianrui_409B | 4.04 | 5.89 | 3.17 | 2.33 |  |  |  |
| HY217 | Japonica | China | Xingguo | 1.15 | - | - | - |  |  |  |
| HY218 | Japonica | Thailand | RD23 | 2.91 | 0.85 | 10.00 | 5.97 |  |  |  |
| HY219 | Indica | China | Mamagu_ | 5.10 | 10.54 | 11.00 | 2.10 |  |  |  |
| HY220 | Indica | China | Meihuanuo | 2.35 | 12.52 | 12.33 | 1.17 |  |  |  |
| HY221 | Japonica | China | Weiguo_ | 0.22 | 1.15 | 11.67 | 0.33 |  |  |  |
| HY222 | Indica | China | Sanbaili | 7.33 | 9.57 | 30.80 | 0.83 |  |  |  |
| HY223 | Japonica | China | Haomake_(K) | 0.24 | 22.76 | 24.16 | 0.33 |  |  |  |
| HY224 | Indica | China | Nangaogu | 3.95 | 10.38 | 10.17 | 1.33 |  |  |  |
| HY225 | Japonica | China | Yuefu | - | 5.48 | 21.50 | 1.17 |  |  |  |

**Supplementary Table S2 | Summary of 56 significantly associated SNPs for our NUE GWAS.**

| Num. | Trait | SNPs | Chr. | Pos(bp) | P value |
| --- | --- | --- | --- | --- | --- |
| 1 | △SV | Chr4_31440122 | 4 | 31440122 | 4.74E-06 |
| 2 | △SV | Chr5_16201470 | 5 | 16201470 | 8.45E-06 |
| 3 | △LL | Chr5_2224399 | 5 | 2224399 | 2.23E-06 |
| 4 | △LL | Chr6_1146665 | 6 | 1146665 | 7.58E-06 |
| 5 | △LL | Chr6_1150436 | 6 | 1150436 | 8.70E-06 |
| 6 | △LL | Chr6_1153064 | 6 | 1153064 | 1.38E-06 |
| 7 | △LL | Chr6_1153228 | 6 | 1153228 | 8.75E-06 |
| 8 | △LL | Chr8_12310229 | 8 | 12310229 | 2.85E-06 |
| 9 | △LL | Chr9_2511615 | 9 | 2511615 | 8.83E-06 |
| 10 | △LL | Chr9_4992111 | 9 | 4992111 | 9.99E-07 |
| 11 | △LL | Chr9_5042096 | 9 | 5042096 | 6.49E-06 |
| 12 | △LL | Chr9_5070032 | 9 | 5070032 | 3.06E-06 |
| 13 | △LL | Chr9_5083309 | 9 | 5083309 | 3.34E-06 |
| 14 | △LL | Chr9_5085419 | 9 | 5085419 | 6.79E-06 |
| 15 | △LL | Chr9_5086546 | 9 | 5086546 | 2.70E-06 |
| 16 | △LL | Chr9_5127355 | 9 | 5127355 | 7.48E-06 |
| 17 | △LL | Chr9_5187526 | 9 | 5187526 | 9.27E-06 |
| 18 | △LL | Chr9_5209212 | 9 | 5209212 | 3.04E-06 |
| 19 | △LL | Chr9_5429573 | 9 | 5429573 | 6.41E-06 |
| 20 | △LL | Chr9_5575159 | 9 | 5575159 | 1.43E-06 |
| 21 | △LL | Chr11_11705315 | 11 | 11705315 | 7.46E-06 |
| 22 | △LL | Chr11_16273119 | 11 | 16273119 | 4.53E-06 |
| 23 | △PH | Chr1_37543388 | 1 | 37543388 | 7.25E-06 |
| 24 | △PH | Chr1_37543388 | 1 | 37543388 | 5.56E-06 |
| 25 | △PH | Chr4_31816339 | 4 | 31816339 | 5.54E-06 |
| 26 | △PH | Chr8_8221884 | 8 | 8221884 | 3.54E-06 |
| 27 | △PH | Chr9_9136610 | 9 | 9136610 | 7.94E-06 |
| 28 | △PH | Chr10_6383686 | 10 | 6383686 | 3.04E-06 |
| 29 | △PH | Chr11_15512919 | 11 | 15512919 | 2.32E-06 |
| 30 | △PH | Chr12_7675038 | 12 | 7675038 | 9.77E-06 |
| 31 | △TN | Chr1_6962638 | 1 | 6962638 | 1.35E-06 |
| 32 | △TN | Chr1_10141302 | 1 | 10141302 | 9.74E-06 |
| 33 | △TN | Chr1_23248523 | 1 | 23248523 | 1.24E-06 |
| 34 | △TN | Chr1_26249482 | 1 | 26249482 | 4.41E-07 |
| 35 | △TN | Chr3_9775291 | 3 | 9775291 | 2.96E-06 |
| 36 | △TN | Chr3_24739518 | 3 | 24739518 | 2.35E-07 |
| 37 | △TN | Chr3_24739537 | 3 | 24739537 | 2.33E-06 |
| 38 | △TN | Chr3_26596148 | 3 | 26596148 | 7.66E-06 |
| 39 | △TN | Chr3_28894227 | 3 | 28894227 | 1.03E-06 |
| 40 | △TN | Chr4_14806662 | 4 | 14806662 | 8.40E-06 |
| 41 | △TN | Chr4_16133552 | 4 | 16133552 | 1.64E-06 |
| 42 | △TN | Chr5_4850031 | 5 | 4850031 | 1.38E-06 |
| 43 | △TN | Chr6_1684791 | 6 | 1684791 | 2.63E-06 |
| 44 | △TN | Chr6_12097334 | 6 | 12097334 | 7.57E-06 |
| 45 | △TN | Chr6_22117430 | 6 | 22117430 | 6.54E-06 |
| 46 | △TN | Chr6_27910816 | 6 | 27910816 | 3.38E-06 |
| 47 | △TN | Chr6_27911777 | 6 | 27911777 | 5.34E-06 |
| 48 | △TN | Chr6_27914235 | 6 | 27914235 | 4.95E-06 |
| 49 | △TN | Chr7_18861905 | 7 | 18861905 | 5.35E-06 |
| 50 | △TN | Chr7_27326210 | 7 | 27326210 | 7.87E-06 |
| 51 | △TN | Chr8_21047943 | 8 | 21047943 | 8.60E-06 |
| 52 | △TN | Chr8_26169335 | 8 | 26169335 | 6.17E-06 |
| 53 | △TN | Chr9_2117574 | 9 | 2117574 | 1.66E-06 |
| 54 | △TN | Chr9_14347361 | 9 | 14347361 | 5.84E-06 |
| 55 | △TN | Chr10_9766608 | 10 | 9766608 | 6.09E-06 |
| 56 | △TN | Chr12_25582237 | 12 | 25582237 | 4.35E-07 |
